# Nociceptive-Evoked Potentials Are Sensitive to Behaviorally Relevant Stimulus Displacements in Egocentric Coordinates

**DOI:** 10.1523/ENEURO.0151-15.2016

**Published:** 2016-07-11

**Authors:** M. Moayedi, G. Di Stefano, M. T. Stubbs, B. Djeugam, M. Liang, G. D. Iannetti

**Affiliations:** 1 Department of Neuroscience, Physiology and Pharmacology, University College London, London WC1E 6BT, United Kingdom; 2 Faculty of Dentistry, University of Toronto, Toronto, M5G 1G6 Canada; 3 Department of Neurology and Psychiatry, Sapienza University of Rome, Rome 00185, Italy; 4 School of Medical Imaging and Tianjin Key Laboratory of Functional Imaging, Tianjin Medical University, Tianjin 300203, China

**Keywords:** nociception, threat detection, attentional reorientation, vertex potential, saliency, body schema, EEG, laser-evoked potentials (LEPs)

## Abstract

Feature selection has been extensively studied in the context of goal-directed behavior, where it is heavily driven by top-down factors. A more primitive version of this function is the detection of bottom-up changes in stimulus features in the environment. Indeed, the nervous system is tuned to detect fast-rising, intense stimuli that are likely to reflect threats, such as nociceptive somatosensory stimuli. These stimuli elicit large brain potentials maximal at the scalp vertex. When elicited by nociceptive laser stimuli, these responses are labeled laser-evoked potentials (LEPs). Although it has been shown that changes in stimulus modality and increases in stimulus intensity evoke large LEPs, it has yet to be determined whether stimulus displacements affect the amplitude of the main LEP waves (N1, N2, and P2). Here, in three experiments, we identified a set of rules that the human nervous system obeys to identify changes in the spatial location of a nociceptive stimulus. We showed that the N2 wave is sensitive to: (1) large displacements between consecutive stimuli in egocentric, but not somatotopic coordinates; and (2) displacements that entail a behaviorally relevant change in the stimulus location. These findings indicate that nociceptive-evoked vertex potentials are sensitive to behaviorally relevant changes in the location of a nociceptive stimulus with respect to the body, and that the hand is a particularly behaviorally important site.

## Significance Statement

The ability to detect behaviorally relevant events when navigating in an ever-changing sensory environment is important for survival. What are the rules that the nervous system obeys to identify which changes in stimulus location are important to attend? Here, we show that changes in the spatial location of a nociceptive stimulus elicit an enhanced brain potential at the scalp vertex (vertex potential) only when they represent a threat to the body. These findings demonstrate that the magnitude of the vertex potential relies on not only low-level stimulus features, but also complex information, such as the stimulus location with respect to the body. Our results support the role of the nociceptive-evoked vertex potential in threat detection in an ever-changing sensory environment.

## Introduction

In a continuously changing sensory environment, the nervous system needs to prioritize features that characterize behaviorally relevant stimuli. The selection of such features has been extensively studied in goal-directed behaviors where it is heavily driven by top-down factors (Fecteau and Munoz, 2006; Chelazzi et al., 2014). A more primitive version of this function is the detection of bottom-up, salient stimulus features (eg, short rise time and high intensity). Interestingly, fast-rising and intense stimuli of various modalities trigger a number of transient responses in the ongoing electroencephalogram (EEG; Walter, 1964). The largest of these consists of a biphasic negative-positive complex, maximal at the scalp vertex (the vertex potential; Bancaud et al., 1953), reflecting multimodal neural activities related to the detection and attentional orientation toward the stimulus (Mouraux and Iannetti, 2009). This response is labeled N1-P2 when elicited by auditory or visual stimuli. In contrast, when elicited by somatosensory stimuli, this response is labeled N2-P2, given that it is preceded by a smaller negative (N1) wave, maximal at the central-temporal regions contralateral to the stimulated hand (Hu et al., 2010; Treede et al., 1988), reflecting early stage sensory processing related to the ascending input (Lee et al., 2009). Nociceptive laser stimuli, which inherently encode potential threats, selectively activate cutaneous nociceptors and consistently elicit a large-amplitude N2-P2 vertex potential related to the activation of Aδ fibres. Notably, the N2 wave elicited by a nociceptive stimulus has been shown to predict defensive motor actions (Moayedi et al., 2015), suggesting that this component may be partly related to the threat content of the stimulus.

When nociceptive stimuli are repeated at a short and constant interval (eg, in a triplet at 1 Hz: S1-S2-S3), the magnitude of the vertex potential elicited by S2 and S3 is significantly smaller than that of S1, ie, a strong habituation occurs (Iannetti et al., 2008). Importantly, this habituation: (1) is not due to neural refractoriness nor to the novelty of the stimulus, but rather to a modulation of stimulus saliency (Wang et al., 2010; Ronga et al., 2013), and (2) is not associated with a similar habituation of the perceived stimulus intensity (Iannetti et al., 2008).

Coupling this triplet paradigm with the systematic manipulation of S3 attributes has proven to be a powerful method to identify which stimulus features are prioritized, and thus modulate the vertex potential amplitude. Importantly, S1 and S2 are identical to ensure that habituation does occur with stimulus repetition; only the S3 attributes are selectively changed. For example, changes of either stimulus modality or stimulus intensity dishabituate the vertex potential, indicating that these features are important determinants of its amplitude (Valentini et al., 2011; Ronga et al., 2013). In contrast, a change of stimulus location from the dorsum of one hand to the other does not modulate the response (Torta et al., 2012). This finding was interpreted as a suggestion that stimulus displacements are less important determinants of vertex potential magnitude. However, this interpretation does not take into account that larger displacements may require greater attentional shifts toward the stimulus, which should be reflected in the vertex potential amplitude. For example, a stimulus displacement from the foot to the hand would trigger an attentional shift and likely elicit a dishabituation of the vertex potential. However, if the foot and the hand were placed next to each other, the displacement in Euclidean (ie, egocentric) coordinates would be small, despite the lack of change in somatotopic distance. In this scenario, the stimulus displacement would not result in a dishabituation of the vertex potential. Here, in Experiment 1, we tested exactly this: whether changes in stimulus location in somatotopic versus egocentric coordinates differentially affect the magnitude of the vertex potential.

Furthermore, displacements of nociceptive stimuli are more likely to signal potentially threatening events when they result in: (1) a decrease of the distance between the stimulus and the core of the body, and (2) the stimulation of important body territories (such as the face and the hand). In Experiments 2 and 3, we aimed to disentangle these two possible explanations.

## Materials and Methods

### Participants

Forty healthy right-handed volunteers took part in the study. Sixteen participated in Experiment 1 [8 females and 8 males, all aged (mean±SD) 25.5 ±6.1 years], 14 in Experiment 2 (6 females and 8 males, all aged 25.3 ±5.2 years) and 14 in Experiment 3 (6 females and 8 males, all aged 23.3 ±3.8 years). All participants gave written informed consent, and the experimental procedures were approved by the local ethics committee.

### Sensory stimuli

Noxious radiant stimuli were generated by two identical infrared neodymium yttrium aluminum perovskite (Nd:YAP) lasers with a wavelength of 1.34 μm (Electronical Engineering). At this wavelength, laser pulses activate directly the Aδ and C-fiber nociceptive terminals located in the superficial skin layers. The laser beam was transmitted via an optic fiber and its diameter was set at ∼6 mm (∼28 mm^2^) by focusing lenses.

To familiarize subjects with the nociceptive stimulus, a small number of low-energy laser pulses were delivered to a skin area of ∼5 × 5 cm, on two stimulation sites: the dorsum of the right foot and the dorsum of the left hand. The hand and the foot were each stimulated with a different laser. The energy of the stimulus was then adjusted individually, separately for the hand and the foot, to elicit a clear pricking pain sensation related to the activation of Aδ nociceptors (Treede et al., 1995), as follows. A laser pulse of low energy (1 J) was first delivered to the target territory. Participants were required to provide a rating describing the intensity of the pinprick, using a numerical rating scale ranging from 0 (“not pinprick at all”) to 10 (“the worst pinprick imaginable”). The stimulus energy was raised in steps of 0.25 J until a rating of 4/10 was consistently obtained. The energy for the foot and the hand stimulations (Experiments 1–3) were adjusted to elicit similar pain intensity percepts [Experiment 1 (mean ±SD): hand= 3.8 ±0.4 J, foot= 4.1 ±0.6 J; Experiment 2: hand= 3.8 ±0.5 J, foot= 4.1 ±0.6 J; Experiment 3: hand= 3.5 ±0.4 J, foot= 3.8 ±0.5 J].

### Experimental design

#### Experiment 1

**Figure 1.**
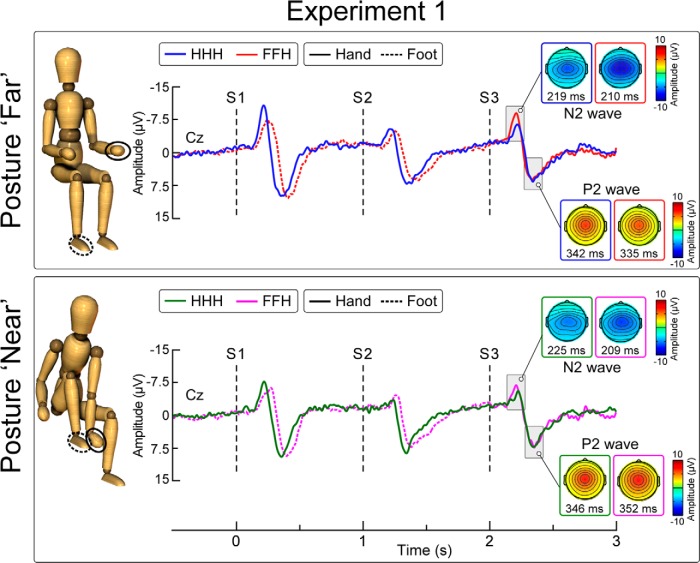
Effect of stimulus location change and posture on laser-evoked vertex potentials (Experiment 1). Group-level average waveforms elicited by trains of three laser stimuli (S1-S2-S3). Displayed signals are recorded from the vertex (Cz, nose reference). S1 and S2 were always delivered on the same body site (hand [H] or foot [F]). S3 was always delivered on the hand. Thus, S3 was delivered either on the same body site as S1 and S2 (HHH, no spatial change) or on a different body site (FFH, spatial change). During the experiment the subject held two postures: far (with the hand and foot ∼100 cm apart, top), and near (with the hand and foot next to each other, ∼10 cm apart, bottom). *x*-axis, time (s); *y*-axis, amplitude (μV). Scalp maps obtained at peak latency of the N2 and P2 waves show the vertex potential elicited by S3.

This experiment consisted of four different blocks of stimulation. In each block, short trains of laser stimuli were presented. Each train consisted of three stimuli (S1**-**S2**-**S3, a triplet) delivered to the dorsum of the foot and/or the dorsum of the hand at a constant interstimuli interval (ISI) of 1 s. This stimulation pattern has been repeatedly demonstrated to yield a robust habituation of corresponding laser-evoked potentials (LEPs; Treede et al., 2003; Iannetti et al., 2008; Lee et al., 2009; Valentini et al., 2011; Torta et al., 2012; Ronga et al., 2013). The time interval between each triplet ranged between 10 and 14 s (rectangular distribution), which is necessary for the S1 of the subsequent trial to elicit an LEP of large amplitude. There were two stimulation patterns: Hand-Hand-Hand (HHH) or Foot-Foot-Hand (FFH). Therefore, S1 and S2 were always on the same site (right foot or left hand), whereas S3 was always on the hand. In two of the four blocks, the subject was sitting, and asked to keep the hand on a table and foot on the floor, resulting in an approximate distance of 100 cm between the stimulated hand and foot (condition “far”). In the other two blocks, participants received HHH and FFH triplets while sitting, but with the hand and the foot placed close to each other on a platform, resulting in an approximate distance of 10 cm between the stimulated hand and the foot (condition “near”; Fig. 1). The order of blocks was counterbalanced across subjects. Before starting the EEG recording, subjects were instructed to relax and equally attend all the stimuli of each triplet, independently of experimental condition or stimulus location.

In each block, we delivered 30 triplets, for a total of 120 triplets in the whole experiment. To avoid nociceptor fatigue or sensitization, between each laser pulse of a given triplet the target of the laser beam was displaced by ∼1 cm along a proximal–distal axis on the hand dorsum. The direction of this displacement was balanced in each block (15 triplets in the proximal direction and 15 triplets in the distal direction). This procedure aimed to minimize differences due to the variation in thickness and innervation of the irradiated skin, and consequently, the variability in the intensity of the somatosensory nociceptive input. Because variations in baseline skin temperature could alter the strength of the nociceptive input (Baumgärtner et al., 2005), an infrared thermometer was used to ensure that baseline skin temperatures were similar at the beginning of each block. At the end of each block, subjects were asked to provide an average rating for the pinprick sensation elicited by each stimulus and each experimental condition, using the same numerical rating scale used in the preliminary procedures to define the laser stimulation energy.

#### Experiment 2

The design of Experiment 2 was exactly the same as Experiment 1, with only two differences. First, stimuli were delivered only in the far body posture. Second, there were four stimulation patterns, as follows. In two of the blocks, they were the same as in the far condition of Experiment 1 (HHH and FFH; ie, the FFH triplets entailed a stimulus displacement toward the core of the body). In the other two blocks, the patterns were FFF and HHF (ie, the HHF triplets entailed a stimulus displacement away from the core of the body).

#### Experiment 3

Stimuli were delivered to the right foot and the left hand, using the same patterns as Experiment 1, ie, HHH and FFH. Stimuli were delivered only in the far body posture, but with the stimulated (left) hand in two alternative positions at the same height: either next to the left side of the trunk, as in Experiments 1 and 2 (condition “near-to-trunk”), or extended out to the left, as far away as possible from the trunk (condition “far-from-trunk”).

### EEG recording

Participants were comfortably seated in a quiet, temperature-controlled room. They were asked to place their left hand and right foot in the positions required by the experimental condition (see above) and to keep their eyes open and gaze slightly downward. EEG was recorded using 32 Ag-AgCl electrodes placed on the scalp according to the International 10-20 system and referenced to the nose. The electro-oculogram (EOG) was recorded from two surface electrodes, one placed over the right lower eyelid, the other placed lateral to the outer canthus of the right eye. Signals were amplified and digitized at a sampling rate of 1024 Hz and a 12-bit conversion, giving a resolution of 0.195 μV (SD32, Micromed).

### EEG analysis

EEG data were preprocessed and analyzed using Letswave 4 (http://nocions.org; Mouraux and Iannetti, 2008) and EEGLAB (Delorme and Makeig, 2004). EEG data were segmented into epochs using a time window ranging from 1 s before S1 to 1 s after the S3 of each triplet (total epoch duration: 4 s). Each epoch was then band-pass filtered from 1 to 30 Hz with a fast-Fourier transform filter. Next, epochs were baseline corrected using the interval of −0.9 s to 0 s before S1 as a reference.

The EOG was used to identify and remove ocular artifacts with a validated method based on independent components analysis (Jung et al., 2000). Independent components related to eye movements had a large EOG channel contribution and a frontal scalp distribution. Epochs with amplitude values exceeding ±100 μV (ie, epochs likely to be contaminated by an artifact) were removed. These epochs constituted 2.3 ± 3.1%, 1.3 ± 1.5%, and 3.6 ± 5.7% of the total number of epochs in Experiments 1, 2, and 3, respectively. Trials belonging to the same experimental condition were aligned to the onset of S1 and averaged. This procedure yielded, for each experiment, four average waveforms per subject, one per condition. We measured, for each subject and condition, the average amplitude of the 20 ms centered on the peak of the N2 and P2 waves (ie, from −10 ms to +10 ms with respect to the peak latency; Luck, 2005). N2 and P2 were recorded at the vertex (Cz), referenced to the nose. The N2 wave was defined as the most negative deflection after stimulus onset and the P2 as the most positive deflection after stimulus onset. Because of the difficulty in isolating the N1 wave in the responses elicited by foot stimulation (Valentini et al., 2012; Hu et al., 2014), the possible modulation of this component was only explored in Experiment 1. The optimal approach to detect the N1 wave elicited by a somatosensory stimulus delivered to the hand is the use of the bipolar montage Cc-Fz (ie, the central electrode contralateral to the stimulated hand referenced to Fz). This montage effectively removes the contribution of the N2 wave to the N1 waveform, because the N2 wave is characterized by an isopotential at electrodes Cc and Fz (Hu et al., 2010). Therefore, the N1 was measured from the central electrode contralateral to the stimulated side (C4), referenced to Fz. It was defined as the negative deflection preceding the N2 wave, which appears as a positive deflection in this montage. Consistent with the analysis of the N2 and P2 waves, the amplitude of the N1 wave was also measured using the average amplitude of the 20 ms around the peak (Luck, 2005).

### Statistical analysis

Statistical analyses are detailed in Table 1, to which superscript letters used in the results refer to. For all three experiments, both psychophysical and electrophysiological data were tested for normality using the Shapiro–Wilk test.

#### Psychophysics

To ensure that intensity ratings of the sensation elicited by laser stimulation were similar across conditions, stimulated limb, and stimuli composing the triplet, we performed a three-way repeated-measures MANOVA for each experiment, with a main factor of “stimulus repetition” (three levels: S1, S2, and S3), and two additional main factors reflecting condition and stimulated limb (with labels specific to each experiment, as listed below). We used a MANOVA because, in all three experiments, the ratings had unequal variances (*p*<0.05).

#### Brain potentials

Given that the somatotopic location of the stimulus affects both the amplitude and the latency of the laser-evoked vertex potential because of differences in peripheral conduction distance, even when perception is matched (Treede et al., 1988; Valentini et al., 2012), all comparisons were performed using responses elicited by stimuli delivered to the same body part.

To confirm that stimulus repetition habituated the LEP response (see Iannetti et al., 2008) we performed paired *t* tests to compare the amplitude of the N2-P2 peaks elicited by S1 and S2, for each experimental condition and experiment.

In Experiment 1, we performed a two-way repeated-measures ANOVA to explore the effect of “posture” (two levels: near, far) and “spatial change” (two levels: “yes”, “no”), as well as their interaction, on the N1, N2, and P2 amplitudes of the S3 response. In Experiment 2, we performed a two-way repeated-measures ANOVA to investigate the effect of “target limb” (two levels: hand, foot) and “spatial change” (two levels: yes, no), as well as their interaction, on the N2 and P2 amplitudes of the S3 response. In Experiment 3, we performed a two-way repeated-measures ANOVA to investigate the effect of “hand position” (two levels: near-to-trunk, far-from-trunk) and “spatial change” (two levels: yes, no), as well as their interaction, on the N2 and P2 amplitudes of the S3 response. Statistical comparisons were performed using SPSS v21 (for peak analysis; IBM).

To explore the spatial distribution of these experimental effects, we also performed the same two-way repeated-measures ANOVAs across all scalp electrodes. Given the peak latency differences across conditions, amplitude values at each electrode were extracted at the latency of the peak of interest (ie, N1, N2, and P2) for each condition and subject. Statistical comparisons across the scalp were performed using MATLAB.

## Results

Table 1 provides information about the data structure, the statistical test and the power for each test described herein. Each statistical test is denoted by a subscript letter.

### Experiment 1

#### Quality and intensity of perception

In all subjects, laser stimuli elicited a clear pinprick sensation, related to the activation of Aδ fibers (Bromm and Treede, 1984). There was no main effect of stimulus repetition, spatial change, and posture on the intensity of the pinprick sensation elicited by the laser stimuli, nor any interaction between these factors.^a^ This indicates that similar intensities of sensation were elicited by the three stimuli of the triplet, following hand and foot stimulation, both in the near and far conditions.

#### Brain potentials

Figure 1 shows the group-level average waveforms in the four conditions. In all conditions, the peak-to-peak amplitude of the N2-P2 wave was significantly larger in S1 than in S2 (all *p* values <0.05).^b–e^

**Figure 2.**
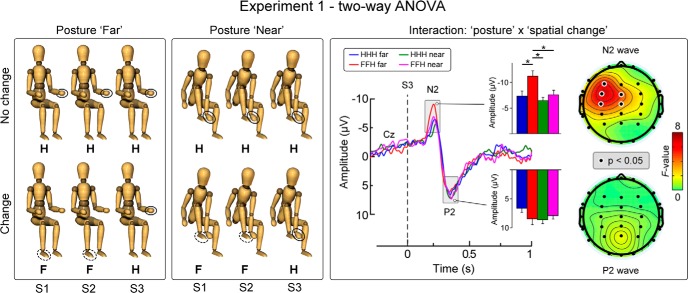
Effect of changes in posture and stimulus location on the laser-evoked vertex potential (Experiment 1). Left, Schematic of the 2 × 2 design used. Right, Statistical effects of the interaction for posture and spatial change on the S3 response, assessed using a repeated-measures ANOVA performed on each subject’s average waveform at the vertex (Cz; nose reference). Bar graphs represent group-level N2 and P2 wave amplitudes (mean ±SE). Scalp maps show the repeated-measures ANOVA results (*F* values) across all recorded electrodes, for the N2 and P2 waves. *p<0.05

We observed a significant main effect of the factor posture on the amplitude of the N1 and N2 waves elicited by S3 (N1: *F*_(1,15)_ = 5.40, *p* = 0.035^f^; N2: *F*_(1,15)_ = 7.74, *p* = 0.014^g^; Fig. 2). There was also a significant main effect of the factor spatial change (*F*_(1,15)_ = 20.18, *p*<0.001^g^) on the amplitude of N2 wave elicited by S3 (Fig. 2). Critically, we found a significant posture × spatial change interaction on the amplitude of the N1 and N2 waves elicited by S3 (N1: *F*_(1,15)_ = 4.63, *p* = 0.048^f^; N2: *F*_(1,15)_ = 7.58, *p* = 0.015^g^; Fig. 2). This interaction indicates that the N1 and N2 were dishabituated only when the somatotopic change occurred while the participant held the far posture (N1 (mean ±SE, hereafter): HHH = −2.7 ±0.41 μV, FFH = −3.6 ±0.57 μV; two-tailed paired *t* test: *t = * 2.13, *p* = 0.05^h^; N2: HHH = −7.3 ±1.0 μV, FFH = −11.1 ±1.1 μV; *t = * 4.78, *p* = 0.0002^i^), but not the near posture (N1: HHH = −2.7 ±0.33 μV, FFH = −2.5 ±0.34 μV; *t = * 0.84, *p* = 0.41^j^; N2: HHH = −6.4 ±0.6 μV, FFH = −7.5 ±0.87 μV; *t = * 1.69, *p* = 0.112^k^). The scalp distributions of this posture × spatial change interaction are shown in Figure 2. There were no significant main effects or interactions on the P2 wave elicited by S3^l^. ANOVA main effects and interactions are summarized in Table 2.

**Table 2.** Summary of ANOVA results (Experiment 1)

			*F*	*p*
N1 wave				
	Main effect of posture		**5.40**	**0.035**
	Main effect of spatial change		2.75	0.344
	Interaction between posture and spatial change		**4.63**	**0.048**
N2 wave				
	Main effect of posture		**7.74**	**0.014**
	Main effect of spatial change		**20.18**	**<0.001**
	Interaction between posture and spatial change		**7.58**	**0.015**
P2 wave				
	Main effect of posture		1.31	0.270
	Main effect of spatial change		0.68	0.424
	Interaction between posture and spatial change		4.08	0.062

Significant effects (*p*<0.05) are highlighted in bold.

### Experiment 2

#### Quality and intensity of perception

In all subjects, laser stimuli elicited a clear pinprick sensation. There was a main effect of spatial change (*F*_(1,13)_ = 11.69, *p* = 0.005), and no main effect of stimulus repetition or target limb, nor any interaction between these factors^m^. The main effect of spatial change indicates that, overall, stimuli delivered in the conditions FFF and HHH (6.3 ±0.4) were perceived as more intense than stimuli delivered in the conditions HHF and FFH (6.1 ±0.4; two-tailed paired *t* test: *t* = 3.42, *p* = 0.005)^n^.

#### Brain potentials

**Figure 3.**
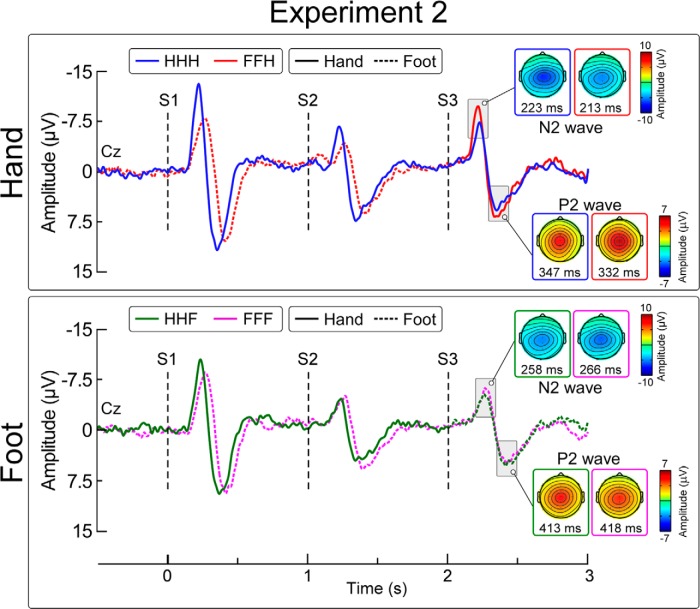
Effect of stimulus location change and target limb on vertex potentials (Experiment 2). Group-level average waveforms elicited by S1, S2, and S3. Displayed signals are recorded from the vertex (Cz, nose reference). S1 and S2 were always delivered on the same body site (hand or foot). In the hand condition (top), S3 was delivered on the hand. Thus, when S3 was on a different body site than S1 and S2 (ie, in triplets FFH) the stimulation pattern produced a spatial progression of the stimulus toward the core of the body. In the foot condition (bottom), S3 was always on the foot. Thus, when S3 was on a different body site than S1 and S2 (ie, in triplets HHF) the stimulation pattern produced a spatial progression of the stimulus away from the core of the body. *x*-axis, time (s); *y*-axis, amplitude (μV). Scalp maps obtained at peak latency of the N2 and P2 waves show the vertex potential elicited by S3.

Figure 3 shows the group-level average waveforms in the four conditions. In all conditions, the peak-to-peak amplitude of the N2-P2 wave was significantly larger in S1 than in S2 (all *p* values <0.05)^o–r^.

**Figure 4.**
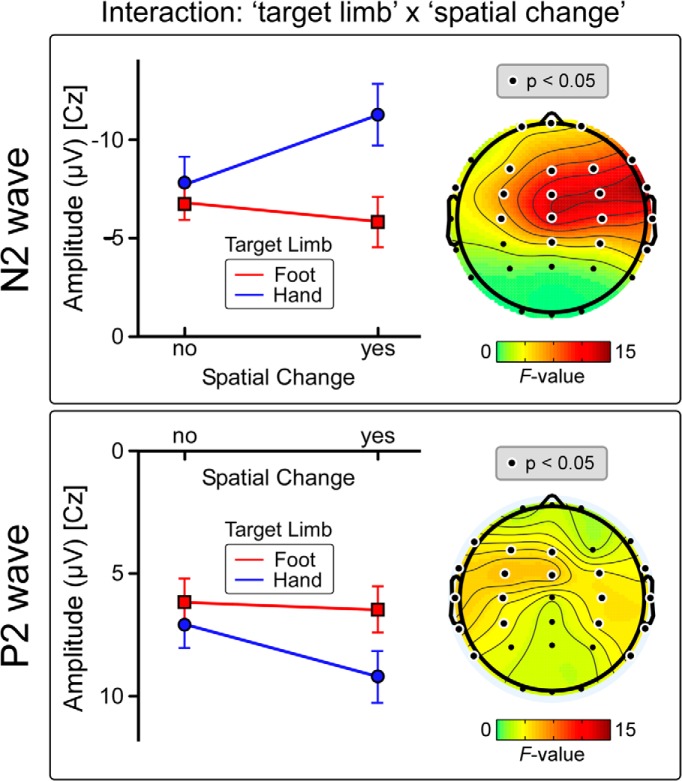
Significant interaction between the effects of stimulus location change and target limb on the N2 amplitude (Experiment 2). The N2 amplitude of the S3 response was assessed using a repeated-measures ANOVA performed on each subject’s average waveform at the vertex (Cz; nose reference). There was a significant target limb × spatial change interaction (*F*_(1,11)_ = 13.89, *p* = 0.003), indicating that the N2 was significantly dishabituated only when the stimulus displacement produced a spatial progression toward the core of the body. Graph data represent mean ±SEM. Scalp maps show the repeated-measures ANOVA results (*F* values) across all electrodes, for the N2 and P2 waves.

We observed a main effect of the factor spatial change on the amplitude of the N2 wave (*F*_(1,13)_ = 20.07, *p* = 0.001^s^), but not of the P2 wave (*F*_(1,13)_ = 2.55, *p* = 0.13^t^) elicited by S3. We also observed a main effect of target limb on the amplitude of the P2 wave (*F*_(1,13)_ = 5.66, *p* = 0.03^t^), but not of the N2 wave (*F*_(1,13)_ = 4.49, *p* = 0.054^s^). Crucially, there was a significant target limb × spatial change interaction on the amplitude of the N2 wave (*F*_(1,13)_ = 13.89, *p* = 0.003^s^; Fig. 4), but not of the P2 wave (*F*_(1,13)_ = 0.22, *p* = 0.64^t^). This interaction indicates that the N2 was significantly dishabituated only when the stimulus was displaced from the foot to the hand (FFH = −11.3 ±1.5 μV, HHH = −7.8 ±1.3 μV; two-tailed paired *t* test: *t = * 4.77, *p*< 0.00004^u^), whereas it was not dishabituated when the stimulus was displaced from the hand to the foot (HHF = −6.8 ±0.9 μV, FFF = −6.5 ±0.9 μV; *t* = 0,282, *p* = 0.78^v^; Fig. 4). The scalp distribution of this posture × spatial change interaction is shown in Figure 4. ANOVA main effects and interactions are summarized in Table 3.

**Table 3.** Summary of ANOVA results (Experiment 2)

			*F*	*p*
N2 wave				
	Main effect of target limb		4.49	0.054
	**Main effect of spatial change**		**20.07**	**0.001**
	**Interaction between target limb and spatial change**		**13.89**	**0.003**
P2 wave				
	**Main effect of target limb**		**5.66**	**0.03**
	Main effect of spatial change		2.55	0.13
	Interaction between target limb and spatial change		0.22	0.64

Significant effects (*p*<0.05) are highlighted in bold.

### Experiment 3

#### Quality and intensity of perception

In all subjects, laser stimuli elicited a clear pinprick sensation. There was no main effect of stimulus repetition, spatial change, and posture on the intensity of the pinprick sensation elicited by the laser stimuli, nor any interaction between these factors^w^. This indicates that the intensity of the sensation was similar in the three stimuli of the triplet, following hand and foot stimulation, both in the near-to-trunk and far-from-trunk conditions.

#### Brain potentials

**Figure 5.**
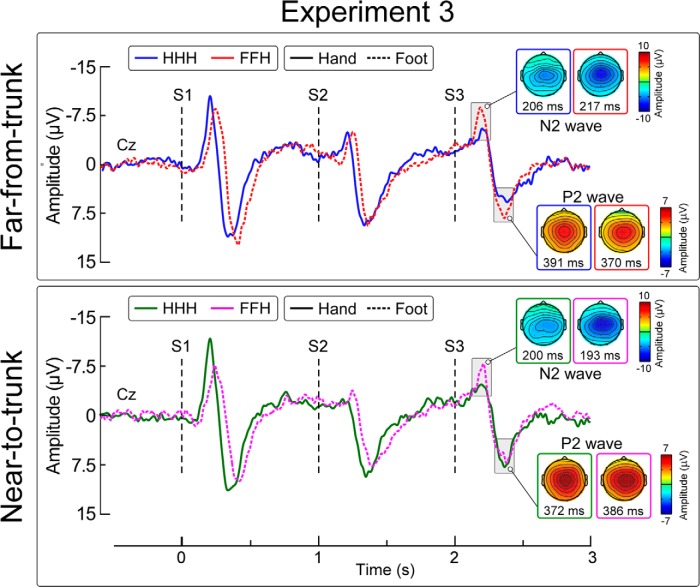
Effect of stimulus location change and hand position on vertex potentials (Experiment 3). Group-level average waveforms elicited by S1, S2, and S3. Displayed signals are recorded from the vertex (Cz, nose reference). S1 and S2 were always delivered on the same body site (hand or foot). S3 was always delivered on the hand. Thus, S3 was delivered either on the same body site as S1 and S2 (HHH, no spatial change) or on a different body site (FFH, spatial change). During the experiment the subject held their hand in two alternative positions at the same height: either next to the left side of the trunk (condition near-to-trunk), or extended out to the left, as far away as possible from the trunk (condition far-from-trunk). *x*-axis, time (s); *y*-axis, amplitude (μV). Scalp maps obtained at peak latency of the N2 and P2 waves show the vertex potential elicited by S3.

Figure 5 shows the group-level average waveforms in the four conditions. The peak-to-peak amplitude of the N2-P2 wave was significantly larger in S1 than in S2, in all four conditions (all *p* values <0.05)^x–aa^.

We observed significant main effects of the factor spatial change (*F*_(1,13)_ = 14.4, *p* = 0.002^bb^) and the factor hand position (*F*_(1,13)_ = 0.51, *p* = 0.487^bb^) on the amplitude of N2 wave elicited by S3, but no interaction (*F*_(1,13)_ = 2.62, *p* = 0.13^bb^). This indicates that the N2 was significantly dishabituated by a stimulus displacement from the foot to the hand, regardless of the position of the hand with respect to the trunk (HHH Far = −7.5 ±0.8 μV, FFH Far = −10.3 ±1.3 μV; two-tailed paired *t* test: *t = * 2.67, *p* = 0.019^cc^; HHH Near = −7.6 ±1.5 μV, FFH Near = 12.5 ±2.3 μV; *t = * 3.75, *p* = 0.002^dd^). There were no significant main effects or interactions for the P2 wave^ee^. ANOVA main effects and interactions are summarized in Table 4.

**Table 4.** Summary of ANOVA results (Experiment 3)

			*F*	*p*
N2 wave				
	Main effect of hand position		0.51	0.487
	Main effect of spatial change		**14.4**	**0.002**
	Interaction between target limb and spatial change		2.62	0.129
P2 wave				
	Main effect of hand position		2.80	0.118
	Main effect of spatial change		3.03	0.105
	Interaction between target limb and spatial change		0.11	0.741

Significant effects (*p*<0.05) are highlighted in bold.

## Discussion

The ability to detect behaviorally relevant events when navigating in an ever-changing sensory environment is important for survival. What are the rules that the nervous system obeys to identify which spatial changes are important to attend to?

Here, we addressed this question by exploring whether and how changes in the spatial location of a stream of otherwise identical nociceptive somatosensory stimuli affect the corresponding brain responses. We observed two main findings. First, the N2 wave of the laser-evoked vertex potential clearly dishabituated when there was a change in stimulus location from the foot to the hand. However, this was not the case when the participant assumed a posture where the stimulated hand and foot were placed next to each other, ie, when the location of the stimulus was similar in egocentric coordinates (Experiment 1). Second, the N2 wave was not dishabituated when the stimulus was displaced from the hand to the foot, but only when it was displaced from the foot to the hand (Experiment 2), and regardless of the distance of the hand from the trunk (Experiment 3). These findings indicate that the nervous system is tuned to detect relevant changes in stimulus location in egocentric coordinates.

### Egocentric, not somatotopic, changes in stimulus location are reflected in vertex potential amplitude

In Experiment 1, we tested whether somatotopic and egocentric changes in stimulus location differentially modulate the amplitude of the vertex potential. We changed the stimulus location from the foot to the hand, while they were either very close (condition near) or separated by ∼100 cm (condition far) in egocentric coordinates (Fig. 1). We found that increasing the somatotopic distance between two stimuli is not sufficient to dishabituate the vertex potential. Indeed, a change in stimulus location from the foot to the hand did not elicit a clear dishabituation when the Euclidean distance between the foot and the hand was minimal (Fig. 2). Rather, the observation that the response was clearly dishabituated when the foot and the hand were further apart demonstrates that the Euclidean distance between the stimuli is a key determinant of its amplitude.

**Table 1.** Statistical tables

Lines	Data structure	Type of test	Power^1^
a	Normal distribution	RM-MANOVA	0.021
b (FFH far)	Normal distribution	Paired *t* test	7.95; 4.20, 11.69
c (FFH near)	Normal distribution	Paired *t* test	4.23; 0.75, 7.70
d (HHH far)	Normal distribution	Paired *t* test	10.07; 5.97, 14.18
e (HHH near)	Normal distribution	Paired *t* test	6.68; 3.22, 10.14
f	Normal distribution	RM-ANOVA	0.236
g	Normal distribution	RM-ANOVA	0.336
h	Normal distribution	Paired *t* test	−0.84; −1.67, 0.0082
i	Normal distribution	Paired *t* test	−3.86; −5.59, −2.14
j	Normal distribution	Paired *t* test	−0.16; −0.25, 0.56
k	Normal distribution	Paired *t* test	−1.14; −2.58, 0.30
l	Normal distribution	RM-ANOVA	0.214
m	Normal distribution	RM-MANOVA	0.129
n	Normal distribution	Paired *t* test	0.26; 0.10, 0.43
o (FFF)	Non-normal distribution	Wilcoxon signed rank test	0.98
p (FFH)	Non-normal distribution	Wilcoxon signed rank test	0.88
q (HHF)	Normal distribution	Paired *t* test	11.61; 6.26, 16.97
r (HHH)	Normal distribution	Paired *t* test	12.57; 7.78, 17.36
s	Normal distribution	RM-ANOVA	0.515
t	Approximate normal distribution^2^	RM-ANOVA	0.017
u	Normal distribution	Paired *t* test	−3.4; −4.99, 1.88
v	Normal distribution	Paired *t* test	0.12; −0.83, 1.1
w	Normal distribution	RM-MANOVA	0.034
x (FFH far)	Normal distribution	Paired *t* test	7.42; 1.11, 13.75
y (FFH near)	Normal distribution	Paired *t* test	9.02; 3.35, 14.69
z (HHH far)	Normal distribution	Paired *t* test	8.23; 1.93, 14.52
aa (HHH near)	Normal distribution	Paired *t* test	13.57; 3.99, 23.16
bb	Normal distribution	RM-ANOVA	0.168
cc	Normal distribution	Paired *t* test	−2.88; −5.22, −0.55
dd	Normal distribution	Paired *t* test	−4.88; −7.69, −2.07
ee	Normal distribution	RM-ANOVA	0.009

1 Power for *t* tests is shown as: mean difference, lower bound of 95% confidence interval, upper bound of 95% confidence interval; Power for RM-ANOVA is partial η^2^ of interaction of interest; Power for Wilcoxon signed rank test is test statistic/rank (W/S).

2 RM-ANOVA is robust against normality violations, and thus only requires approximate normal distribution. Only the P2 HHH condition violated the normality assumption based on the Shapiro–Wilks test.

Notably, the N2 component was clearly modulated by the distance between the hand and the foot in egocentric coordinates, while the P2 component was not. This is an important finding that confirms that the neural activities contributing to these components, as well as the stimulus information content to which they are sensitive, are heterogeneous (Garcia-Larrea et al., 1997; Lee et al., 2009). Our finding indicates that the amplitude of the N2 wave does not simply reflect changes in stimulus location in somatotopic coordinates. Indeed, the stimulus location on the skin was not different between conditions (the S3 was always directed to the dorsum of the hand in all conditions of Experiment 1). Rather, the key factor determining the N2 amplitude in Experiment 1 was the change of the location of the nociceptive stimulus in external space. Such mapping of stimulus location in egocentric coordinates requires integrating the somatosensory information about where the stimulus was delivered on the skin with the information about the position of the stimulated body part in space; the so-called “body schema” (Serino and Haggard, 2010).

The observation that the vertex potential is not sensitive to changes of stimulus location in somatotopic, but is sensitive to changes in egocentric coordinates, clearly indicates that the neural activities underlying this response are related to changes in behaviorally meaningful information about stimulus location. This highlights the survival value of the vertex potential. Indeed, the response is elicited only by a novel stimulus that is important to detect and react to appropriately.

### Stimulus displacements toward important body parts are reflected in vertex potential amplitude

Given the proposition that the vertex potential is related to the detection and appropriate reaction to behaviorally relevant stimuli (Iannetti and Mouraux, 2010; Ronga et al., 2013), we hypothesized that the direction of the change in stimulus location with respect to the core of the body, in addition to its absolute Euclidean distance, is an important determinant of the vertex potential. In Experiment 2, we observed that only changes in stimulus location entailing a spatial progression toward the core of the body (ie, from the foot to the hand) elicited a response dishabituation, whereas in stimulus location entailing a spatial progression away from the core of the body (ie, from the hand to the foot) did not (Figs. 3, 4). This observation shows that large displacements are not sufficient to dishabituate a vertex potential, as the distance between foot and hand was the same in both conditions. Furthermore, nociceptive stimuli are inherently threatening (Melzack and Wall, 2004), and that the trial-by-trial variability in amplitude of the N2 wave of the laser-evoked potential better encodes defensive than non-defensive motor responses (Moayedi et al, 2015). For all these reasons, we propose that the N2 modulation observed in the current experiment was determined by the threat content of the stimulus. Indeed, changes in spatial location in egocentric coordinates that bring a potential threat stimulus closer to the body signal a potentially greater danger. Importantly, this interpretation does not preclude the possibility that non-threatening but still behaviorally relevant stimuli may also determine the amplitude of the negative wave of the vertex potential.

An additional interpretation is that the N2 dishabituation is caused not only by stimulus displacements toward the core of the body, but also toward an important body part such as the hand. To disentangle these two possible explanations, we performed a third experiment where subjects held their hand in two positions: either near to or far from the trunk. If the magnitude of the N2 is determined by a displacement toward the core of the body, then the N2 would dishabituate in the near-to-trunk condition, but not in the far-from-trunk condition. However, if the N2 would also dishabituate in the far-from-trunk condition, it would indicate that the N2 is also sensitive to the stimulation of body parts that are important to defend. The observation that the N2 wave was dishabituated regardless of the distance between the hand and the trunk (Fig. 5) supports the latter explanation.

External frames of reference are typically centered on important body parts, such as the face and the hand. These frames of reference are formed by proprioceptive and visual information (Ho and Spence, 2007; Medina and Rapp, 2008), and are used to judge the distance of environmental stimuli relative to these body parts (de Vignemont et al., 2005). Additionally, there is evidence that somatotopic information about threatening stimuli, such as noxious stimuli, is integrated with visual information in both frontal and parietal multimodal neurons that code for the spatial location of these stimuli with respect to these body parts (Dong et al., 1994; Graziano et al., 1997). Notably, these neurons fire maximally when the threatening stimuli move along a trajectory toward their receptive fields (Rizzolatti et al., 1981; Colby et al., 1993; Graziano et al., 1997). Therefore, rather than simply encoding stimulus location in somatotopic coordinates, the nervous system uses the information about stimulus location within this external frame of reference to make predictions about the spatial location of forthcoming sensory input. If: (1) the location of the subsequent sensory stimulus violates the prediction, and (2) its location is closer to a behaviorally important body part, then a vertex potential of enhanced magnitude is elicited.

The results of Experiments 2 and 3 might seem at odds with those of Experiment 1. Indeed, in the near condition of Experiment 1, the stimulus displacement from the foot to the hand did not elicit a dishabituated response. It is important to note, however, that in this condition the hand and the foot are close to each other in egocentric coordinates. Therefore, stimulus features are hierarchically prioritized; the egocentric distance between two consecutive stimuli is likely to be a more important determinant of the N2 amplitude than the importance of the hand. This hierarchical set of rules suggested by this finding is further discussed below.

This further explains the lack of a dishabituation of the N2 observed when the location of stimulation is changed from the dorsum of one hand to the other, when the hands are placed ∼40 cm from each other and at the same distance from the body (Torta et al., 2012). In this scenario, the Euclidean distance between the two stimuli is small. There is evidence from behavioral studies that visual attention is not necessarily captured by novel sensory stimuli, but rather by transient features of novel stimuli such as direction of motion (Franconeri and Simons, 2003; Franconeri et al., 2005; Hollingworth et al., 2010). Our data suggest that the vertex potential is not simply determined by the transient nature of the stimulus, in line with previous findings. For example, only increases in stimulus intensity elicit a vertex potential of larger amplitude (Ronga et al., 2013). Therefore, there is a set of hierarchical rules by which the nervous system detects a sensory stimulus is threatening, and consequently, a vertex potential occurs.

### The laser-evoked N2 wave encodes potentially threatening stimuli based on a set of rules

We have demonstrated that the N2 wave of the laser-evoked vertex potential is determined by the information gathered by mapping the spatial location of the eliciting stimulus in egocentric coordinates (Figs. 1, 2). More specifically, we have shown that the N2 wave is sensitive to changes in Euclidean, not somatotopic distance, and that these changes must entail a behaviorally relevant change in the stimulus location (Figs. 3–5). Together with previous findings, we can therefore develop a heuristic model of the stimulus features that determine the amplitude of the somatosensory N2 wave. When identical stimuli are delivered in a stream at short and constant ISI the vertex potential strongly habituates (Fruhstorfer, 1971; Iannetti et al., 2008), and selectively changing the modality or increasing the intensity of one of these stimuli yields a strong response dishabituation (Valentini et al., 2011; Ronga et al., 2013). Notably, only changes in spatial location that: (1) entailed an increase in Euclidean distance (Fig. 2), and (2) resulted in the stimulation of an important body part (Figs. 4, 5) clearly dishabituated the somatosensory N2 wave.

Together, these results provide compelling evidence that vertex potentials elicited by nociceptive stimuli are sensitive to specific changes in stimulus features. These changes must indicate that the stimulus has a greater behavioral relevance compared with previous stimuli of the same sensory modality. These findings provide further evidence that the vertex potential is therefore related to the detection and the appropriate reaction to behaviorally relevant stimulus changes in the sensory environment.

## Synthesis

The decision was a result of the Reviewing Editor Lila Davachi and the peer reviewers coming together and discussing their recommendations until a consensus was reached. A fact-based synthesis statement explaining their decision and outlining what is needed to prepare a revision is listed below. The following reviewer(s) agreed to reveal their identity: John Kramer

Reviewer 1:

The manuscript reports on the effects of body position and nociceptive evoked potentials. This is an interesting study that builds on recent literature in the field on the importance of peripersonal body space and nociceptive processing. The study was well designed and the analyses used were appropriate. The findings are straightforward: experiments 1 and 2 together clearly shows an effect of spatial change on N2 amplitude and latency that is dependent on the distal to proximal shift of the stimulus. There is no effect on P2. N1 and N2 amplitudes were also affected by posture alone. The manuscript is very clearly written. Overall this is a good study that makes a valuable contribution to the literature.

A possible concern is that there is something special about processing pain in the hand vs foot. A stimulus to the hand might be more relevant and worth protecting than the foot, regardless of its position relative to the core of the body. Possible solutions to this problem would be to stimulate the core itself or to switch the relative distance of the foot and hand from the core, in which case the HHF change should have an effect similar to FFH in the current study.

There is no discussion of the main effect of posture on N2 and N1. This should be added.

Reviewer 2:

This is an interesting paper that examines the relative contribution of the limb position to the generation of prominent laser evoked potential (LEP) waveforms. In two separate experiments, using a "triplet" stimulation paradigm, the authors test LEPs in the hands and feet (dorsum), as well as manipulate body position (Experiment 1 only). In Experiment 1, 3 consecutive stimuli in the hand led to an apparent reduction in LEP amplitude (N2 and P2 waveforms). Two consecutive stimuli in foot (1-2), followed by one stimuli applied in the hand (3) was shown to "dishabituate" the reduction of the third stimulus. In response to the same stimulation pattern, dishabituation was not evident when the hand was moved closer to the foot (i.e., posture 'near', same 'egocentric coordinates). In Experiment 2, subjects maintained a 'far' posture. Interestingly, only when the stimulus location moved from "distal-to-proximal" was dishabituation of N2 observed. Comparatively, there was no dishabitutation when moved from "proximal-to-distal" (i.e., hand-hand-foot). The authors conclude the LEPs are sensitive to "changes in stimulus location in egocentric coordinates", as well as "changes results in in a reduction of the distance between the stimulus and the core of the body". The manuscript is well written and the methods are thoroughly explained. The results are clearly described and illustrated in Figures.

This reviewer has two major concerns:

The statistical comparison was solely focused on S3. To this reviewer, this is a significant shortcoming of the statistical design. This becomes problematic in that dishabituation is a relative term, but the statistics are not making the comparison to S1 or S2, only within S3 (to the understanding of this reviewer). On one hand, the advantage of this approach is that the same stimulation area is used for comparison (hand). However, in this reviewer's opinion, repeated measures ANOVA could be implemented to examine main and interaction effects in the different posture conditions ('far' and 'near') and stimulation paradigms (HHH and FFH). In principle, this should confirm habituation in the HHH condition (significant reductions in amplitude from S1 to S3, in both the 'far' and 'near' postures), and dishabituation related to FFH stimulation (increased amplitude for S3, 'far' posture ONLY).

However, what is most confusing to this reviewer: would not the ideal experiment involve the same subjects participating in all conditions (Experiments 1 and 2), manipulating the distance of the hand relative to the midline? Experiments 1 and 2 appear, to some extent, needlessly complex. Did the authors consider a condition whereby the arm and hand was moved away from the trunk? According to their theory, moving the hand further away from the trunk should inhibit dishabituation caused by FFH (as understood by this reviewer). In some ways, this would represent the most straightforward evidence in support of their conclusions, and is readily testable.

Minor comments:

The authors conclude that the vertex potential is not sensitive to changes in stimulus location in somatotopic coordinates. Some caution is warranted here. This conclusion should only hold true if peripheral distance is held constant. Stimulating the shoulder or the trunk compared to the hand has the effect of increasing afferent synchronization and reducing temporal dispersion, as well as any effect due to distal-to-proximal changes.

It would be helpful to the reader to understand how the data shown here is different than studies that have addressed differences in LEPs/CHEPs based on different stimulation locations. There are clear differences, but if the conclusion is that somatotopic coordinates do not influence amplitudes, this will contrast (in some reader's minds) with what is known (for example, that shoulder and trunk stimulation yield larger evoked potentials, which has been shown with EEG and fMRI).

For this reviewer, it is somewhat difficult to reconcile that the hand is "proximal" relative to the foot. Anatomically speaking, both the hand and the foot are "distal". Could other terms be used?
